# Supplementary figures and images for: N-6-methyladenosine (m6A) promotes the nuclear retention of mRNAs with intact 5′ splice site motifs
Source: Life Sci Alliance. 2024 Dec 3;8(2):e202403142. doi: 10.26508/lsa.202403142 (PMC11629677; doi:10.26508/lsa.202403142)

**B**

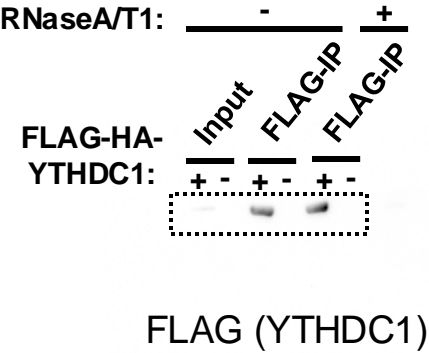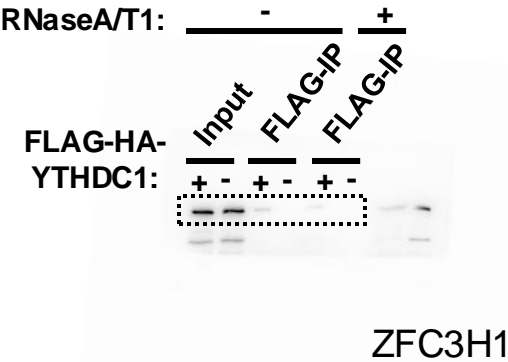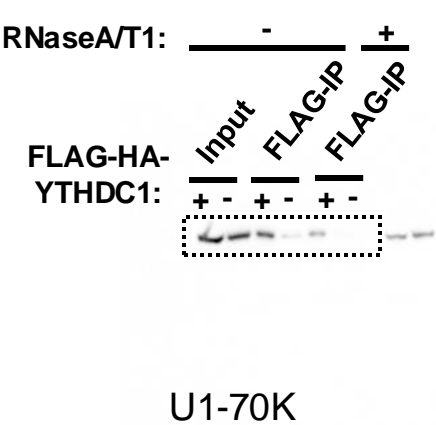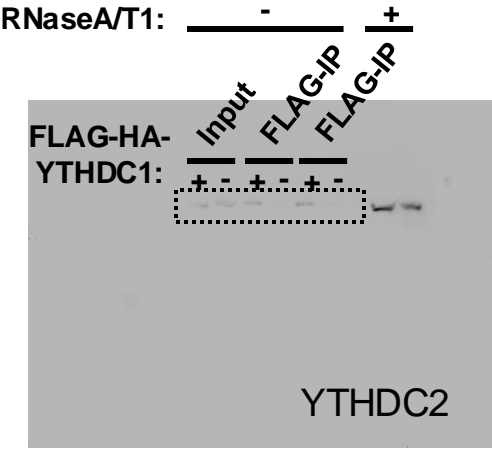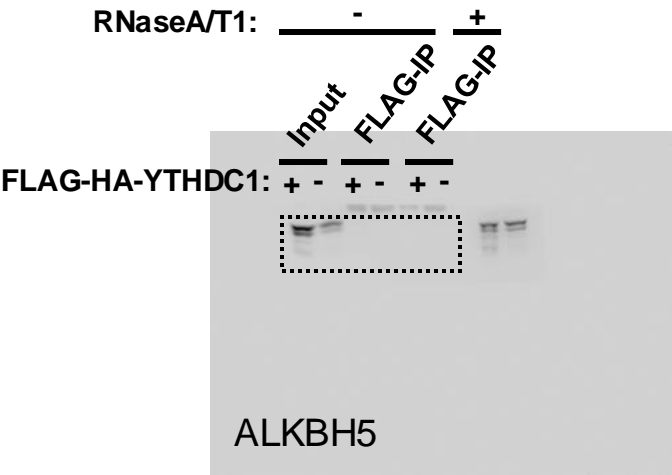

C

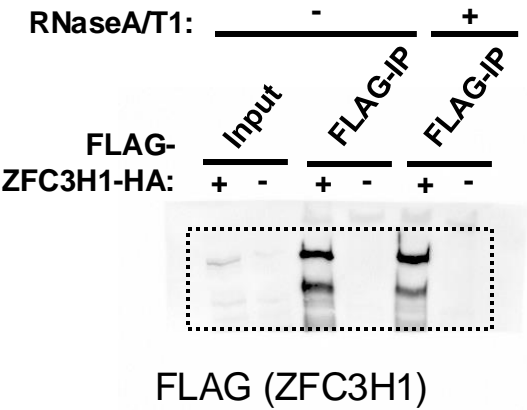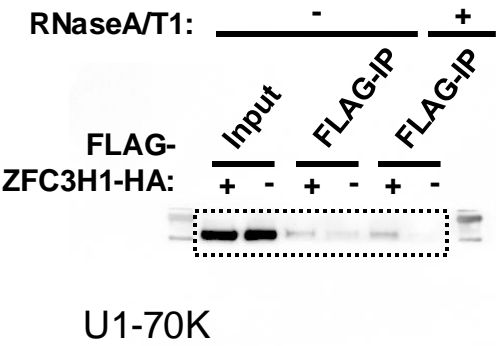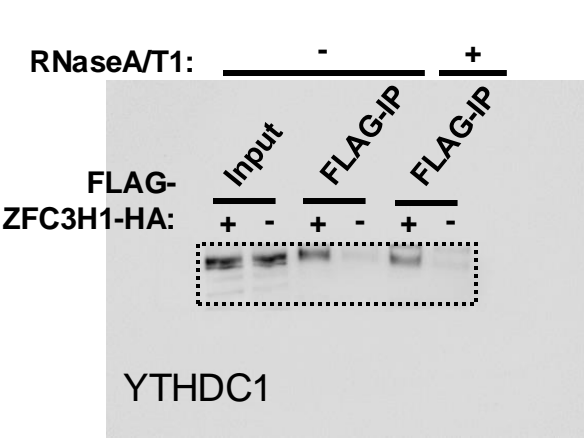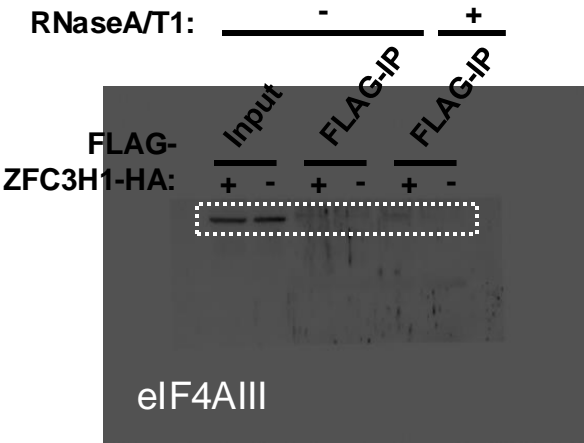

D

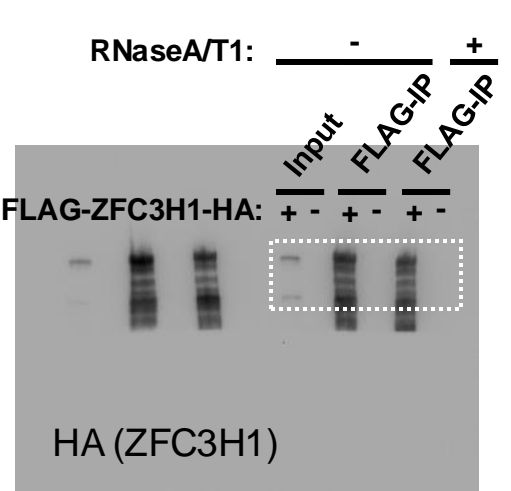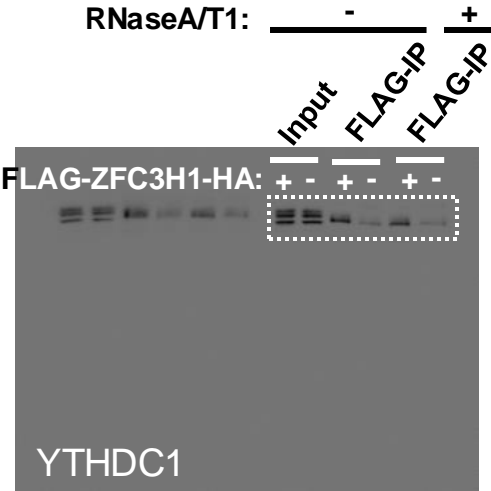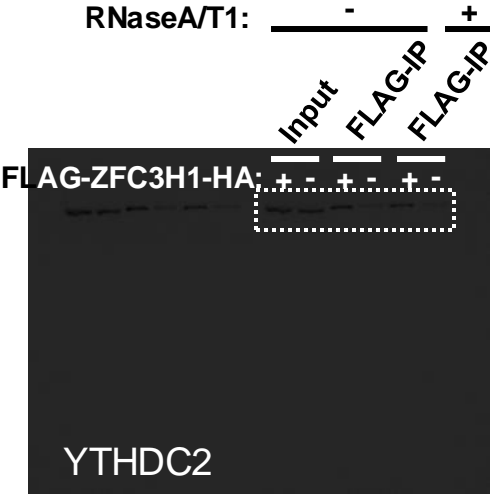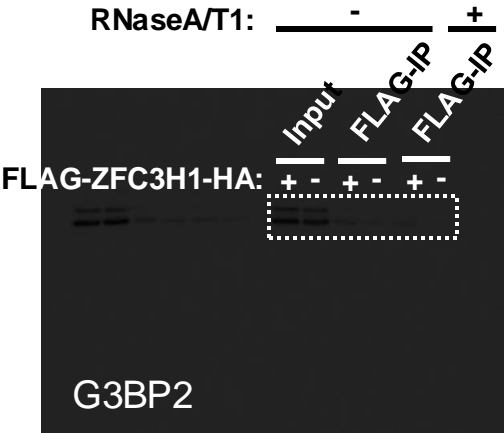

E

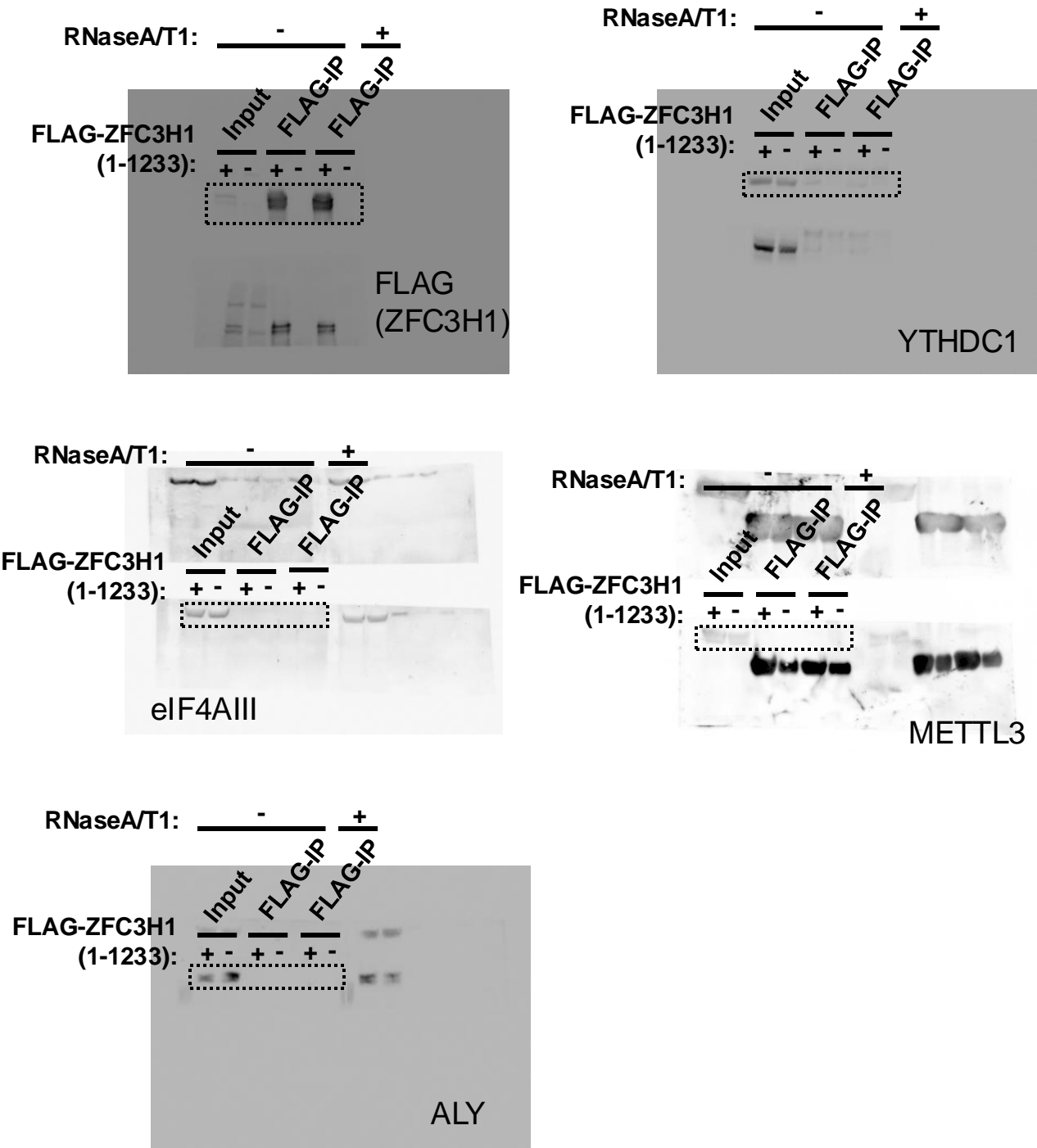

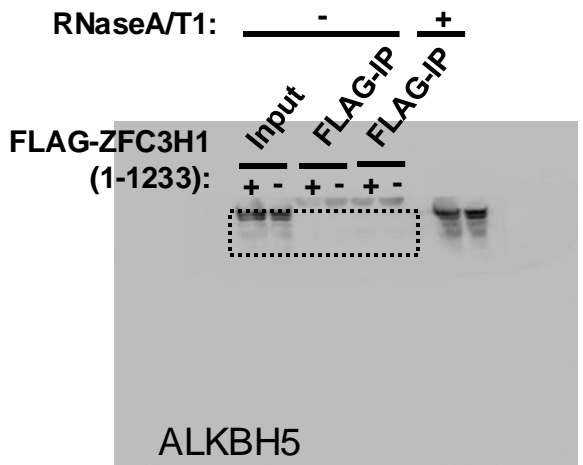

Supplement: Supplementary file 1 [file LSA-2024-03142_SdataF1.pdf]

A

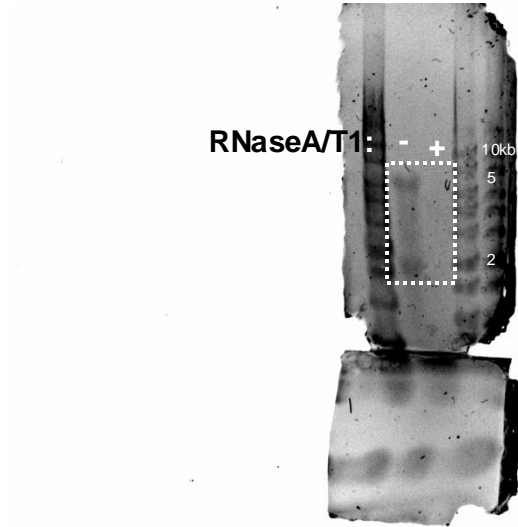

D

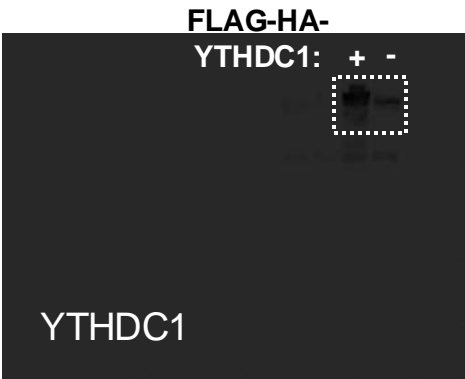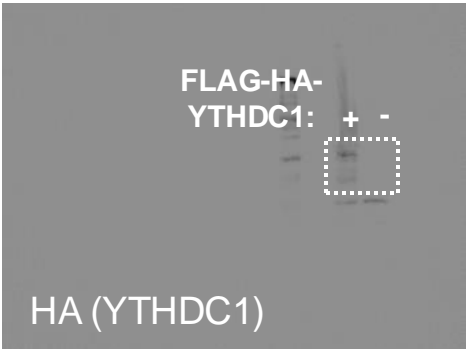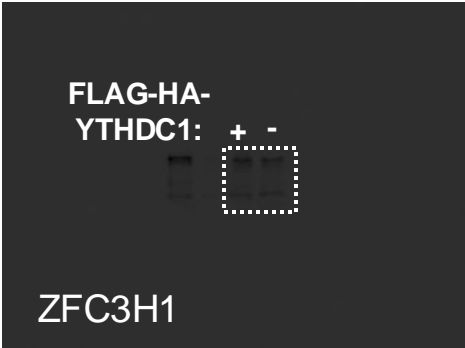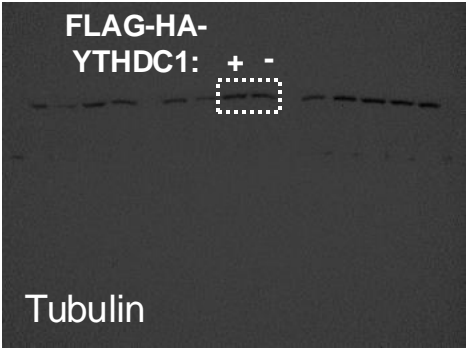

G

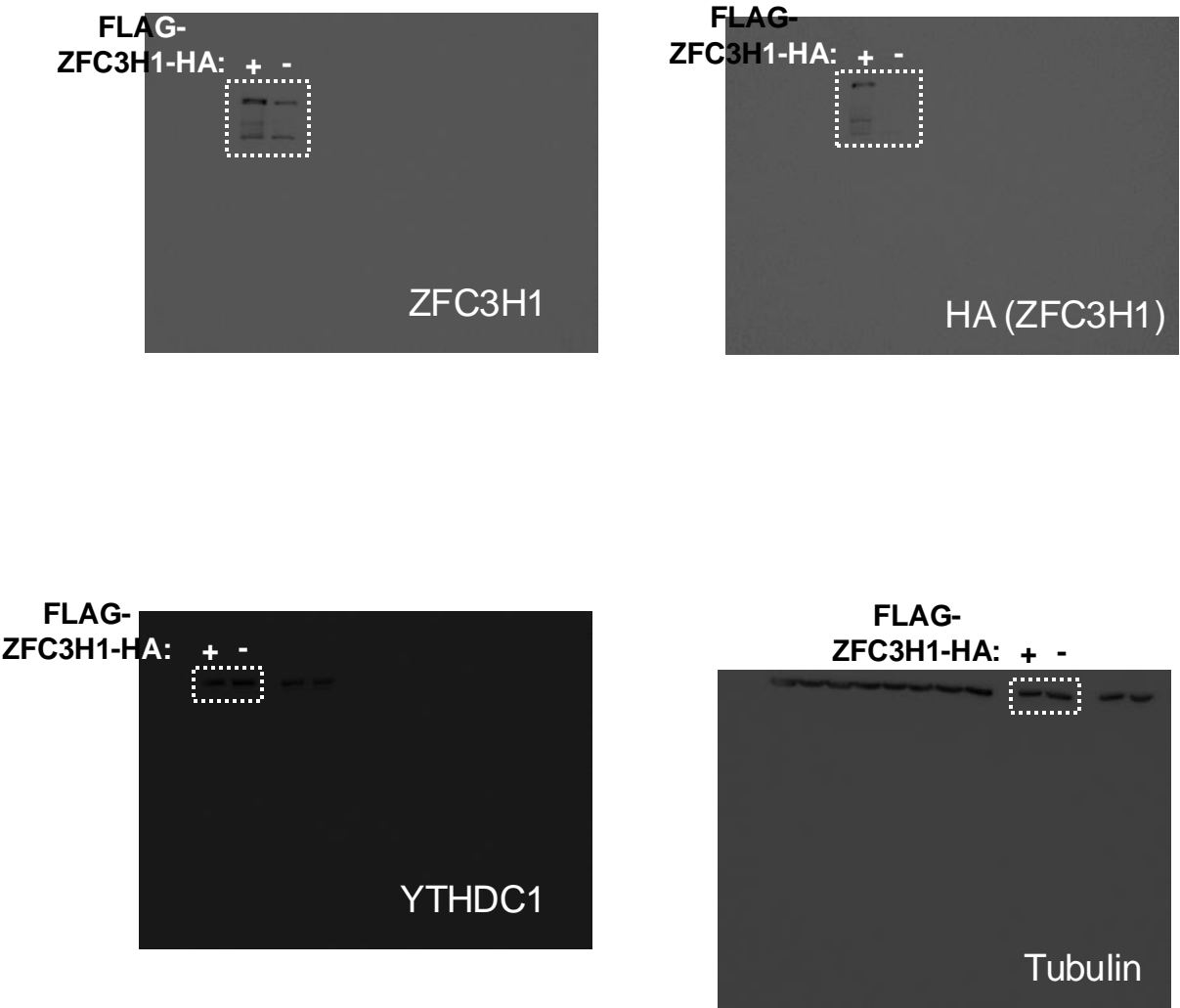

Supplement: Supplementary file 2 [file LSA-2024-03142_SdataFS1.pdf]

G

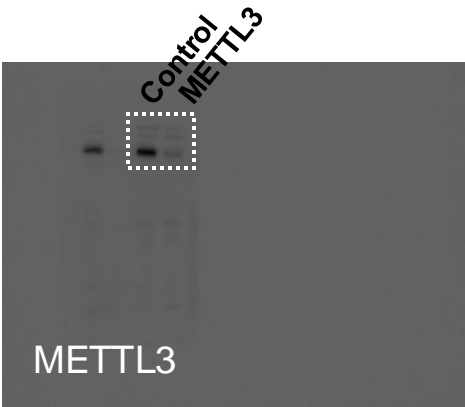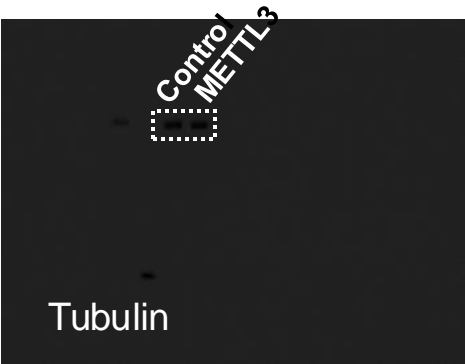

Supplement: Supplementary file 3 [file LSA-2024-03142_SdataF2.pdf]

A

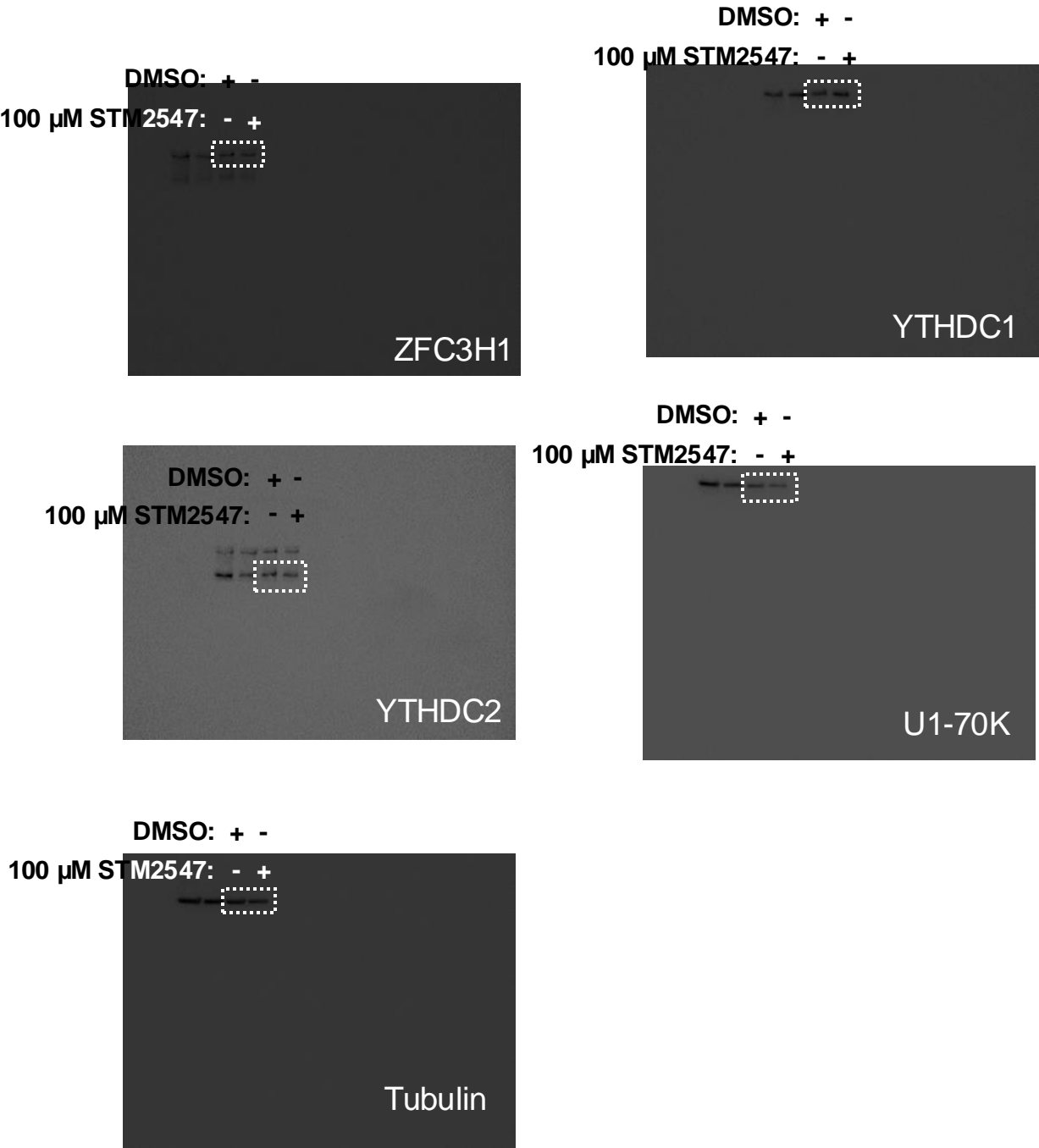

Supplement: Supplementary file 4 [file LSA-2024-03142_SdataFS2.pdf]

**A**

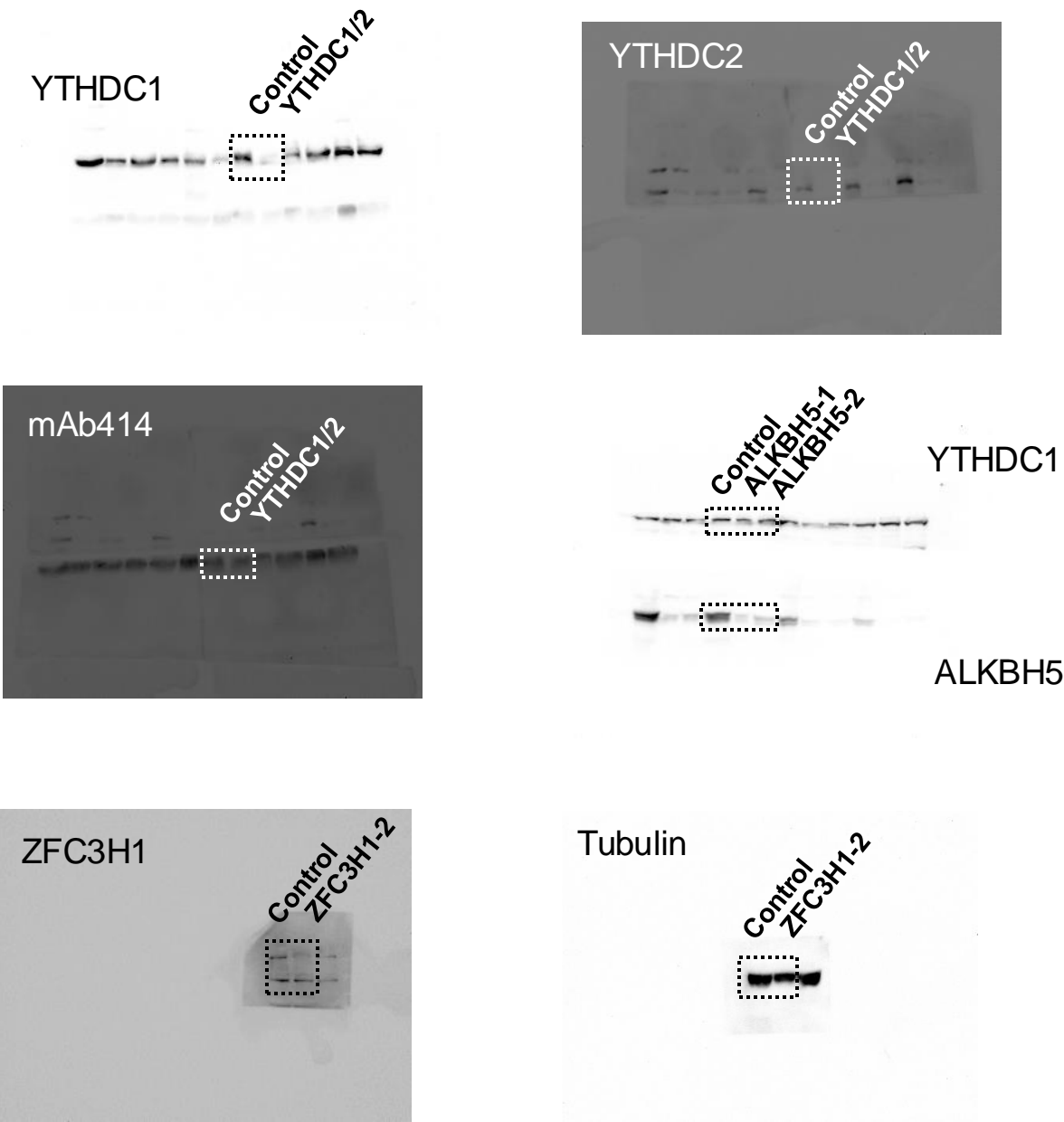

Supplement: Supplementary file 5 [file LSA-2024-03142_SdataF3.pdf]

A

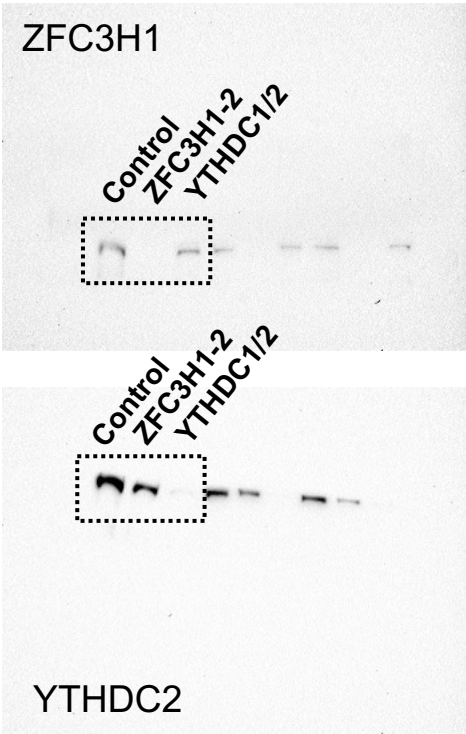

YTHDC1

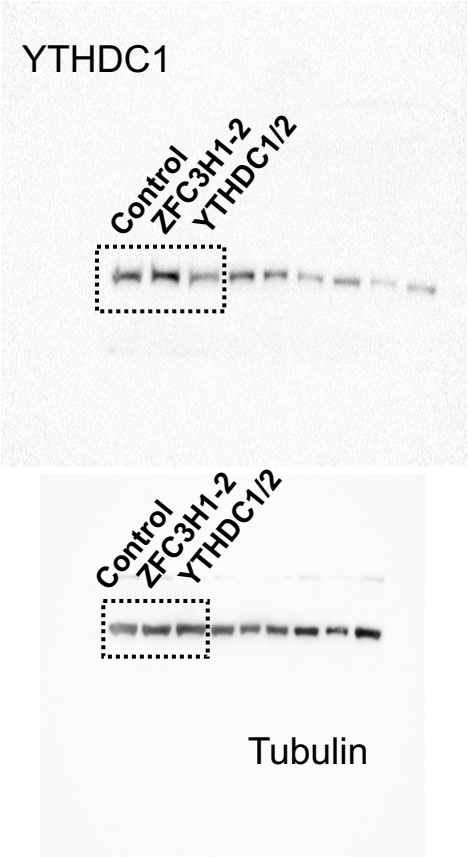

F

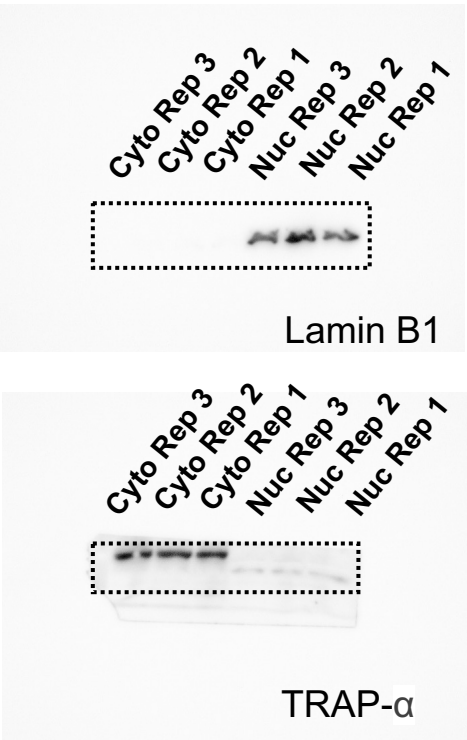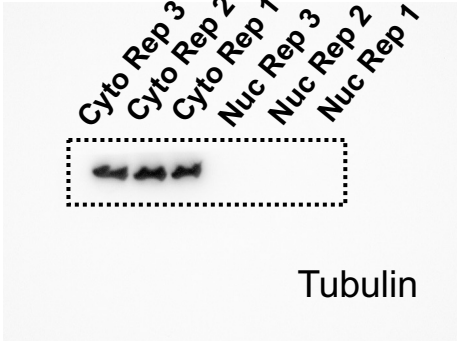

**G**

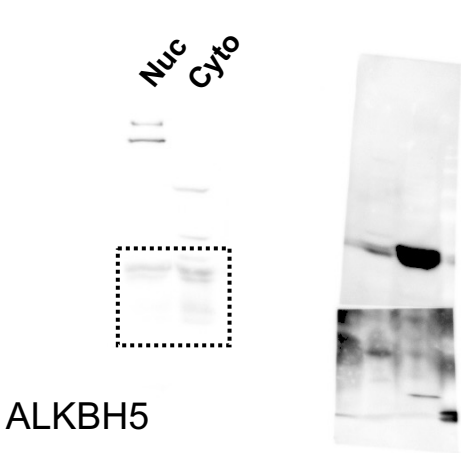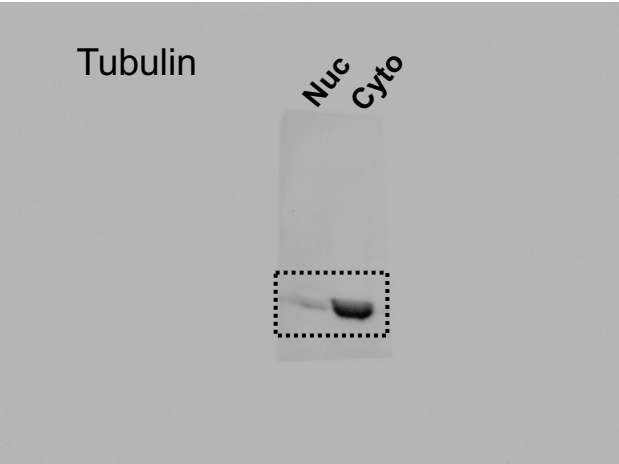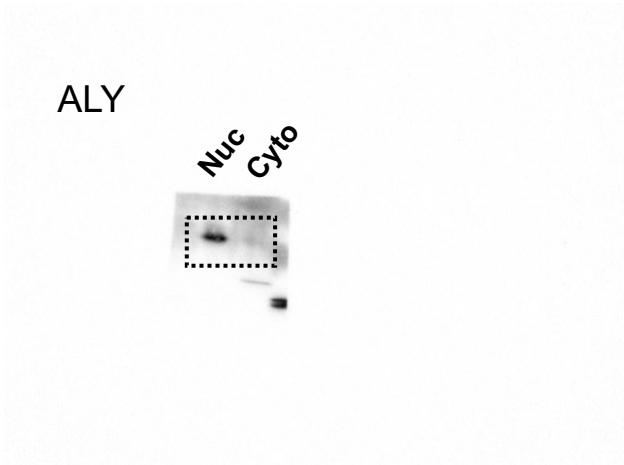

Supplement: Supplementary file 6 [file LSA-2024-03142_SdataFS3.pdf]
